# Q&A: The novel coronavirus outbreak causing COVID-19

**DOI:** 10.1186/s12916-020-01533-w

**Published:** 2020-02-28

**Authors:** Dale Fisher, David Heymann

**Affiliations:** 1 Division of infectious Diseases, University Medicine Cluster, National University Health Systems, Singapore, Singapore; 2 Yong Loo Lin School of Medicine, National University of Singapore, Singapore, Singapore; 3 London School of Hygiene & Tropical Medicine, London, UK

## What is COVID-19, and what do we know so far about its clinical presentation?

The virus responsible for COVID-19, SARS-CoV-2, is in the species SARS-like corona viruses. At 125 nm, it is slightly larger than influenza, SARS and MERS viruses. It is almost certainly a descendant from a bat corona virus of which there are many. The closest is a virus that originated from the Rhinolophus bat which is > 96% homologous with the current SARS-CoV-2 virus. It is only 79% homologous with the original SARS CoV [1].

The near identical gene sequences of 90 analysed cases from outside of China suggests it has likely emerged after a solitary species jump in early December 2019 from an unknown (likely mammalian) intermediate host [2]. Pangolins are an endangered ant-eating mammal from which scientists in Guangzhou have shown a coronavirus with 99% homology, with a receptor binding domain identical to that of SARS-CoV-2. However, this has not been confirmed, and, in addition, the pangolin's rarity means this may not be the only mammal involved.

The symptoms of COVID-19 are fever, dry cough, fatigue, nasal congestion, sore throat and diarrhoea. On February 14th, the Chinese Center for Disease Control and Prevention (China CDC) published the first details of 44,672 confirmed cases, in the biggest study since the outbreak began [3]. Their findings show that COVID-19 was mild for 81% of patients and had an overall case fatality rate of 2.3%. Of those confirmed cases, only 2.2% were under 20 years old. Compared to adults, children generally present with much milder clinical symptoms. It is likely that future serological studies will show much asymptomatic disease in children. As opposed to H1N1, pregnant women do not appear to be at higher risk of severe disease. The severity of the disease appears to be associated with age, with the elderly most at risk; those over 80 years of age had a Case Fatality Rate (CFR) of 14.8%. The CFR was also increased in those with comorbidities including cardiovascular, diabetes, chronic respiratory disease, hypertension, and cancer. The cause of death is respiratory failure, shock or multiple organ failure.

## How are infected people being treated?

There is no proven treatment at this early stage but we will doubtless have more information about this soon. It can be assumed that non-pharmacologic approaches are effective such as fluid support, oxygen and ventilatory support. Most recently the national data suggests that 17.7%, 10.4% and 7.0% of all cases have disease requiring respiratory support in Wuhan, Hubei (not including Wuhan) and the rest of China (respectively). About a quarter of all require ventilation while 75% require oxygen support only. The variation in severity rates probably reflects the outcomes in an overwhelmed health system. Extra Corporeal Membrane Oxygenation (ECMO) is potentially of benefit and we will know more particularly when cities with higher technology health systems become affected and ECMO is truly tested in the most severely ill. ECMO is currently being used in China, however, its effectiveness is yet to be determined.

Antiviral drugs as well as a variety of other putative treatments are typically being prescribed for deteriorating patients on a compassionate basis. Clinicians would be well aware of such situations but assurance is required that their safety and efficacy are being scientifically assessed so that meaning is brought to bear quickly. Coordination of clinical trials to avoid duplication and ensure that results are rapidly available will be a challenge but the case numbers should facilitate rapid definitive results. (see What is in the pipeline for vaccine development and/or therapeutics?).

## Why is the World Health Organization (WHO) so concerned about it?

As a novel virus newly emerged in humans, the world’s population is completely immune-naïve and therefore vulnerable. There is clear human-to-human transmission in family clusters in China and beyond, transmission from close face-to-face social contact, especially in small enclosed spaces, and transmission from failed infection prevention and control measures in health facilities. In addition, the experience in Wuhan shows that transmission can be massive in a short period of time with thousands of new patients diagnosed daily.

The current aim of the global response is to flatten the epidemic curve so that transmission is slowed, and to interrupt transmission where possible. While there is clearly a mortality linked to the virus, the most concerning problem will be if a health system is overwhelmed in the wake of rapid transmission so that affected patients cannot receive the care they need. Furthermore, patients with other urgent medical conditions are at risk of not obtaining their necessary care. Countries with vulnerable health systems are particularly of concern.

Recently, there have been outbreaks in newly affected countries including Italy and Iran where the index case is unidentified. Furthermore affected countries have very large clusters emerging such as Korea and Japan. There is no reason to believe that the global community is ready for this emerging pandemic; ready in a way that can see drastic public health intervention implemented within days, including aggressive and massive contact tracing, monitoring (or quarantine) and early detection and isolation in an attempt to slow the progression.

## How are health agencies reacting?

There is uncertainty regarding transmissibility and severity – more information is emerging about the spectrum of disease, especially mild disease, which is not identified using many current case definitions, and about the ease of transmission from person to person. Health agencies are unsure how to model this and estimates vary depending on the variables being used. For instance, SARS was essentially spread later in the disease from patients with more significant clinical pictures, and it was contained by infection control measures particularly in hospitals. The limited spread to family members of health workers and the community was contained by usual outbreak control measures including early identification and management of persons with infection, tracing of contacts with monitoring for onset of fever and/or symptoms, and active engagement of communities.

Most modelling suggests that the severity of illness is more like influenza than SARS, and there is concern among the public health community because the transmissibility of COVID-19 is not yet fully understood, and the potential for it to become endemic like other respiratory pathogens is unknown.

Because of the many unknowns, the initial reaction by health agencies is still valid: to brace for the first wave as the virus reaches a completely naïve population and to make maximum effort to interrupt transmission [4, 5].

Experience with managing this outbreak will be very heterogenous across the world. Countries closely connected with China, such as Singapore, will be ahead in this regard. As the outbreak moves across regions, there is opportunity to support those affected later both in terms of readiness and response. Mechanisms available to outbreak response organisations, particularly through the Global Outbreak Alert and Response Network (GOARN), can be valuable in skills- and knowledge-sharing. Therefore, there should be a deliberate effort to utilise knowledge from early affected countries in later affected countries.

## What measures are likely to be successful in curbing its further spread?

On January 23rd, Hubei province in Central China was locked down with all movement in and out of the province blocked. Travel across China was discouraged and the number of scheduled flights and train journeys available considerably reduced to perhaps 10% of previous activity. Commercial and social activities became negligible, with schools, restaurants, other entertainment spots and most shops closed. Migrant workers were prevented from returning to work after the extended Chinese New Year Holiday. Frequent hand hygiene when in public and staying at home became the norm. This unprecedented public health effort by China has afforded the rest China and the world a lead time to prepare. The lockdown resulted in a downward trend in national and provincial epidemic curves, however, these measures are not sustainable and eventually there will be a strategy to return to normality. Should this result in a second wave of cases, and more international spread, countries around the world must be prepared. Early identification, and isolation or cohorting of positive cases to designated sites is at the core. In order to achieve this, hospitals, quarantine services, laboratories, together with epidemiology and communication teams will need to be scaled up to provide effective and efficient care.

## What is in the pipeline for vaccine development and/or therapeutics?

It appears that no vaccine will be available for at least one year, likely a little longer. Phase 1 trials for safety and immunogenicity in human populations are likely within 3 months.

In terms of therapeutics there is no known effective pharmaceutical agent. There are over 200 registered clinical trials registered in China alone. Putative agents include antivirals; Griffithsin, a spike protein inhibitor, nucleoside analogues eg. remdesivir, ribavirin and protease inhibitors such as lopinavir/ritonavir. Immunomodulatory and other host targeted agents include interferon, chloroquine and immunoglobulins. Corticosteroids will potentially have benefit for immune mediated lung damage late in the course of disease [6]. Much of the theory stems from what we have learnt from limited trials in other corona viruses [7].

## Data Availability

Not Applicable.
